# Near-Infrared Spectroscopy (NIRS) as a Method for Biological Sex Discrimination in the Endangered Houston Toad (*Anaxyrus houstonensis*)

**DOI:** 10.3390/mps5010004

**Published:** 2021-12-30

**Authors:** Li-Dunn Chen, Mariana Santos-Rivera, Isabella J. Burger, Andrew J. Kouba, Diane M. Barber, Carrie K. Vance

**Affiliations:** 1 Department of Biochemistry, Molecular Biology, Entomology, and Plant Pathology, Mississippi State University, Starkville, MS 39762, USA; lc1817@msstate.edu (L.-D.C.); jms2033@msstate.edu (M.S.-R.); 2 Department of Wildlife, Fisheries, and Aquaculture, Mississippi State University, Starkville, MS 39762, USA; id113@msstate.edu (I.J.B.); a.kouba@msstate.edu (A.J.K.); 3 Department of Ectotherms, Fort Worth Zoo, Fort Worth, TX 76110, USA; DBarber@fortworthzoo.org

**Keywords:** amphibians, chemometrics, dimorphism, monomorphism, reflectance spectroscopy

## Abstract

Biological sex is one of the more critically important physiological parameters needed for managing threatened animal species because it is crucial for informing several of the management decisions surrounding conservation breeding programs. Near-infrared spectroscopy (NIRS) is a non-invasive technology that has been recently applied in the field of wildlife science to evaluate various aspects of animal physiology and may have potential as an in vivo technique for determining biological sex in live amphibian species. This study investigated whether NIRS could be used as a rapid and non-invasive method for discriminating biological sex in the endangered Houston toad (*Anaxyrus houstonensis*). NIR spectra (*N* = 396) were collected from live *A. houstonensis* individuals (*N* = 132), and distinct spectral patterns between males and females were identified using chemometrics. Linear discriminant analysis (PCA-LDA) classified the spectra from each biological sex with accuracy ≥ 98% in the calibration and internal validation datasets and 94% in the external validation process. Through the use of NIRS, we have determined that unique spectral signatures can be holistically captured in the skin of male and female anurans, bringing to light the possibility of further application of this technique for juveniles and sexually monomorphic species, whose sex designation is important for breeding-related decisions.

## 1. Introduction

Amphibians are the most threatened group of vertebrate taxa, with over 40% of species showing dramatic population declines and well over 100 species having gone extinct in the last several decades [1]. The loss of amphibian biodiversity epitomizes the current Anthropocene epoch, or sixth mass extinction event [2,3]. The primary contributors to amphibian extirpation include habitat loss, climate change, chemical pollutants, and infectious diseases [4]. In order to mitigate the decline of amphibian populations occurring worldwide, captive assurance colonies have been established at zoological and research institutions across the globe. The ultimate goal of such programs is two-fold: (1) to create genetically diverse and sustainable captive populations, and (2) to recover in situ populations through reintroductions of progeny produced ex situ to their historic habitats. Unfortunately, many captive populations of amphibians are either not sustainable due to low reproductive output or have low gene diversity since not all individuals breed [5]. Although the absence of environmental cues in captive breeding programs is the primary cause for poor reproductive performance [6,7], many amphibian species are monomorphic and sex discrimination can also be a hindrance to pairing animals and establishing appropriate breeding recommendations.

Sex identification is critical for informing management decisions surrounding captive breeding programs, such as breeding pair selection, maintenance of an appropriate sex ratio, avoidance of inbreeding among tank mates, management and transfer of juveniles for the purpose of optimizing genetic diversity, and informing practitioners on what reproductive technologies should be applied for propagating the species of interest. For example, the reproductive hormone regimens (e.g., hCG, human chorionic gonadotropin and GnRH, gonadotropin-releasing hormone) used to induce spermiation in males and ovulation in females are often sex-specific and should therefore be tailored to the individual’s sex [8,9,10]. However, sex classification remains a challenge, particularly for monomorphic species, many dimorphic species outside of the breeding season, and most juvenile amphibians [11,12]. 

While some methods for determining the biological sex of monomorphic species and juveniles have been developed, most of these technologies are costly, labor-intensive, and/or limited in their application. For example, the biological sex of dusky gopher frogs (*Lithobates sevosus*), white-bellied frogs (*Geocrinia alba*), orange-bellied frogs, (*Geocrinia vitellina)*, and Maud Island frogs (*Leiopelma pakeka*) has previously been determined through the assessment of testosterone and estrone ratios via urinary and fecal hormone analyses [13,14,15]. Albeit effective in differentiating biological sex, hormone analysis requires numerous chemical reagents, is costly, and requires substantial time and resources for hormone extraction and validation procedures to conduct the assays [13,14,15]. Additionally, amphibian hormones are often at the limit of detectability for assays that were initially developed for mammalian species, which may make the validation process challenging and potentially unreliable. Biological sex discrimination has also been evaluated using magnetic resonance imaging and ultrasonography, but the former is limited in application to in-house use only, and both require sexually mature individuals that exhibit detectable sex characteristics [13,16]. Furthermore, sex-specific morphological and behavioral differences, such as the presence of thumb pads and enlarged throat sacs used to produce vocalizations in males, vary between species and may not be present outside of the normal breeding season, particularly for juveniles and seasonally monomorphic species [11]. On a molecular level, most species exhibit homomorphic sex chromosomes, making karyotypic analyses unreliable [17]. Although analyses evaluating microsatellites and single-nucleotide polymorphisms to determine genetic sex are becoming increasingly more effective, they require tissue sample collection, genetic markers, substantial cost and time, and expertise related to DNA sequencing [18]. The resulting time lag between sample collection, data analysis, and interpretation of results, in addition to costs, may be disadvantageous in situations where practitioners at captive breeding facilities require on-the-ground, real-time assessments on which to base a decision. 

Near-infrared spectroscopy (NIRS) is a portable, non-destructive, biophotonic tool that functions by measuring the biochemical properties of a given sample. When light energy is applied to a sample, the chemical bonds vibrate at specific wavelengths and frequencies that are characteristic of the chemical constituents comprising the tested material [19]. NIRS measures the vibrational light energy that is either transmitted through or reflected from the sample of interest in real time [20]. NIRS has been used extensively in the agricultural and pharmaceutical industries to ensure quality control of numerous critical process parameters [21,22] (e.g., the chemical composition of plants, drugs, etc.) and has increasingly been used to study animal physiology [23,24]. Unlike traditional single-purpose assays (e.g., hormone assays), NIRS is non-destructive and capable of generating a plethora of valuable multivariate physiochemical information from a single sample [23]. The spectral data obtained from any given sample reflect the complex suite of chemical properties that underlie both the internal and external factors contributing to an animal’s physiology. For example, physiological parameters that allow for species identification, evaluation of hormone profiles and reproductive status, and the ability to distinguish between biological sex within a species have been measured from biotic samples such as feces, urine, and blood [23]. 

The porous nature of amphibian skin allows for the direct application of NIRS technologies in vivo [24]. The skin of amphibians is known to contain characteristics of a fully functioning endocrine gland, such that dermal tissues contain hormone receptors as well as the ability to express hormone activity [25]. We hypothesized that a spectroscopic probe may be placed directly onto the skin of a live amphibian to non-invasively collect information on a suite of naturally occurring chemical compounds expressed by the individual. Chemometric analyses may then be applied to the resulting spectral profiles in order to extract chemical information using an array of mathematical and statistical methods to develop a prediction model for the physiological parameter(s) of interest. Once a reliable prediction model has been established for the parameters, it is possible to collect and analyze future spectral scans instantaneously. NIRS modeling, therefore, has the advantage of providing on-the-ground diagnostics in real time to decision makers in both ex situ and in situ settings. 

The Houston toad (*Anaxyrus houstonensis*) is an endangered anuran species endemic to the plains of Central Texas [26] and was the first U.S. amphibian species to be listed under the Endangered Species Conservation Act [27]. *A. houstonensis* exhibits marked dimorphism during the breeding season, making it possible to assign biological sex to individuals. Males are easily identifiable as they possess nuptial pads and black throat sacs, and produce distinct mating calls during the breeding season, signaled by increased precipitation in the early spring months [28]. Therefore, *A. houstonensis* may serve as a model of known dimorphism to provide a ‘proof of principle’ for NIRS analysis to discriminate sex in a living animal system. Although these sex-specific traits are present both during and outside the breeding months for *A. houstonensis* adults, such traits may be less pronounced or absent in individuals until they reach sexual maturity [29,30]. Therefore, the sexual identification of juveniles cannot rely on visual markers, meaning that NIRS may be useful in sexing juveniles even in sexually dimorphic species. Having an early detection system would facilitate the genetic transfer of animals between breeding institutions, instead of having to wait several years for sexual maturity to be reached when associated morphometric traits become distinguishable or gametes are produced. 

Considering the value of demographic and life history information for informing decisions surrounding animal management and species conservation, it is pertinent to further develop methods where biological sex can be quickly and effectively assessed. The application of NIRS could be particularly useful for species that exhibit a high level of sexual monomorphism, as well as for non-gravid females, juveniles, and individuals outside of the breeding season, when sex-specific traits are diminished or absent. Previous research found that it was possible to detect distinct, sex-specific chemical signals in three salamander species using the spectra collected from their mental and postcloacal glands, which are known to express pheromones (i.e., chemical signals) in excess during the breeding season [31]. NIRS, as a method for sex discrimination, has been applied in anurans [11,23], yet the presence of integument reproductive glands and use of pheromones is not well understood [32]. The goal of the current study was to determine if chemical signals could be detected in the skin of a model anuran species, *A. houstonensis,* through the application of NIRS. Our specific aim was to test NIRS scanning of toad skin, coupled with chemometrics, as an in vivo analytical technique to evaluate biological sex and gravidity. 

## 2. Materials and Methods

Sexually mature *A. houstonensis* individuals from the Fort Worth Zoo (Fort Worth, TX, USA) were selected for this study based on the age and visual assessment of male phenotypic characteristics (e.g., darkened throat patches and nuptial pads) or presence of eggs in females, which was identified through ultrasound imaging [16]. Experiments were approved by and adhered to the guidelines and regulations set forth by the Fort Worth Zoo (IACUC #17-H001; federal permit TE051818-0). Adult *A. houstonensis* were housed in 18” × 26” × 15” containers with up to four same-sex adults in each group. Each enclosure was illuminated with a 10.0 Reptibulb UVB light and placed on a slight incline to create a land–water environment, where a hide box with sphagnum moss comprised the dry side and water was allowed to pool at the bottom. Enclosures were flushed daily with reconstituted reverse osmosis water and cleaned weekly. *A. houstonensis* were seasonally conditioned to promote natural hibernation behavior from October to January by gradually lowering the temperature from room temperature to 13 °C. For the rest of the year, the toads were kept at 28 °C. 

**Figure 1:**
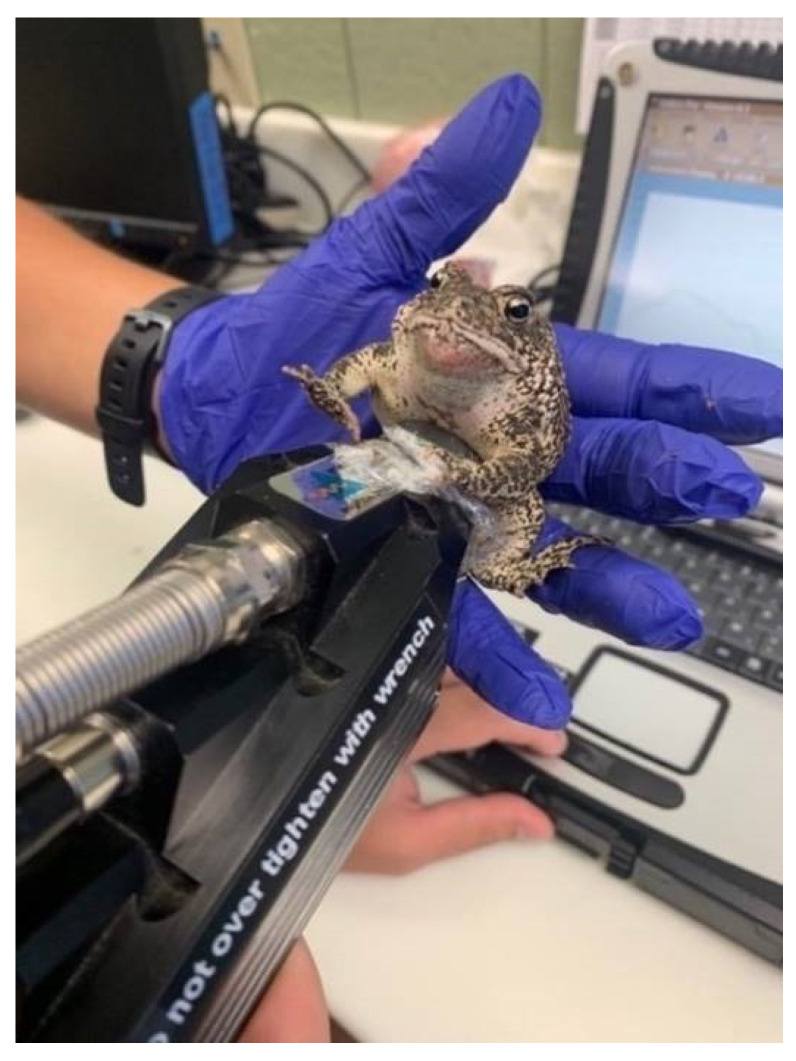
NIR spectra collection from *A. houstonensis* at the Fort Worth Zoo. Spectra were collected superior to the cloaca on the ventral side of the toad. Animals underwent ultrasound imaging immediately following NIR spectra collection and then returned to their enclosures.

In preparation for spectral data collection, plastic wrap was used to cover the probe to avoid biochemical interference between individuals. In order to establish a baseline for reflectance, a white (Spectralon^®^) reference spectrum was captured between each individual. NIR reflectance spectra (λ = 350–2500 nm) were collected from adult females (*N* = 81; mean weight = 59.5 ± 15.9 g) and males (*N* = 51; mean weight = 51.2 ± 8.4 g) using a portable spectrophotometer, the ASD FieldSpec^®^ 3 Indico^®^Pro (Malvern Panalytical, Analytical Spectral Devices Inc., Boulder, CO, USA), and a 2 cm diameter low-powered plant contact probe. Live individuals had their ventral sides blotted dry with a sterile Kimwipe™, and spectral data were collected in triplicate for each individual by placing the contact probe on the abdomen, superior to the cloaca (Figure 1). Each replicate consisted of 50 scans averaged together into a single spectrum with an integration time of 136 ms. Individuals immediately underwent ultrasound imaging and then returned to their enclosures following data collection. All spectra were collected between February and May 2021, which coincided with the normal breeding season for this species [33].

To develop the prediction models, a balanced dataset with equal numbers of spectra from males and females was created by randomly sorting spectra into an 80:20 calibration/validation distribution (CAL/VAL = 216/54 spectra; equivalent to 72/18 individuals) to test for mathematical pre-processing and avoid modeling bias. Chemometrics was applied to the near-infrared range of 700–2000 nm using the program Unscrambler^®^ X v.10.5 (CAMO Analytics, Oslo, Norway). Mathematical pretreatments of Standard Normal Variate (SNV), linear de-trending, and first derivative Savitzky–Golay (symmetrical smoothing points = 8) were applied to the spectral database prior to principal component analysis (PCA) and linear discriminant analysis (PCA-LDA).

**Figure 2:**
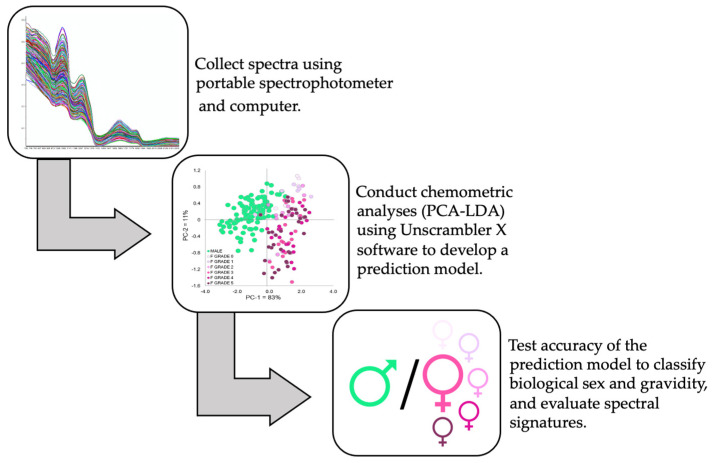
Simple schematic outlining the workflow followed in this study.

PCA was utilized to visualize the trends and outliers present in the spectral database. PCA on the mean-centered matrix was obtained by full (leave-one-out) cross-validation and algorithm-singular value decomposition (SVD). PCA was applied before the LDA to reduce the dimensionality of the spectral database. The PCA-LDA model using the Mahalanobis method was generated with the top-down approach for PC selection. The results from the PCA-LDA calibration are reported in the confusion matrix, and the percent (%) accuracy was calculated to evaluate its performance. The generated PCA-LDA model was tested on the internal validation set and an external validation set containing 36 females and 6 males. Females were assessed by ultrasound immediately after the NIR spectral data were collected, and ultrasound grades were also incorporated post hoc in the PCA scores plot to evaluate females of varying reproductive states with consideration of male scores. Ultrasound data were collected for each female using a 0–5 grading scale adapted from Graham et al. [34], where 0 indicated a lack of follicular development and 5 indicated advanced folliculogenesis with large, fully developed eggs. A simplified schematic of the workflow involved in spectral data collection and analysis is outlined in Figure 2.

## 3. Results 

**Figure 3:**
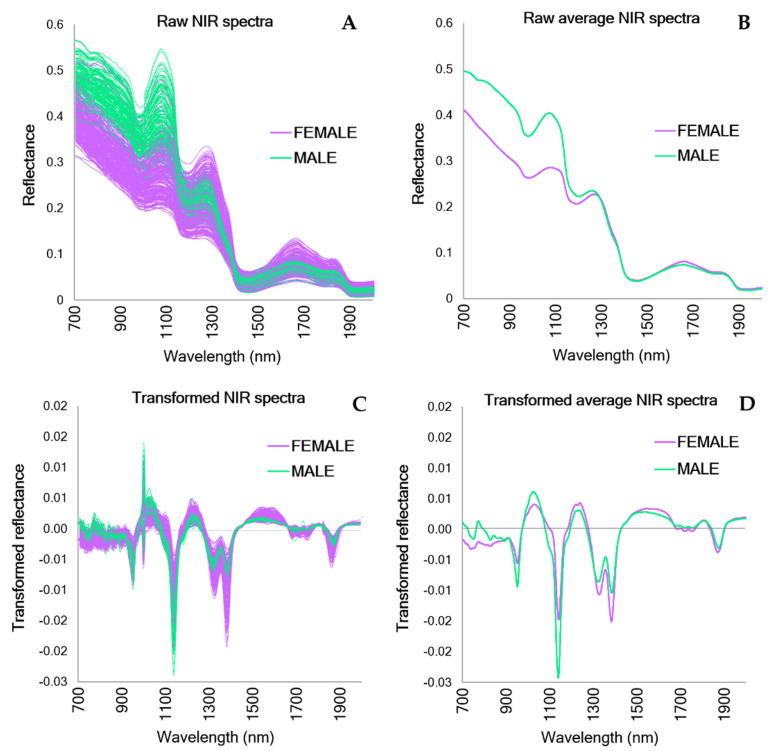
Raw and transformed NIR spectra (700–2000 nm) from male and female *A. houstonensis* individuals indicate distinct spectral patterns between the two biological sexes. (**A**) Raw spectra from the full database (*N* = 396). (**B**) Raw average NIR spectra categorized by biological sex. (**C**) Transformed NIR spectra from the full database (*N* = 396). (**D**) Transformed average NIR spectra across male and female *A. houstonensis* individuals.

Upon inspection of the raw and transformed spectral data, visually distinct patterns differentiating males and females were observed (Figure 3). Major differences in the spectra for males and females can be observed in the raw spectra on the third overtone region of the NIR spectrum between 700 and 1200 nm (Figure 3B). The transformed spectra showed different patterns between sexes in the same vibrational bands as the raw spectra, yet also indicated differences in the 1200–1700 nm range, which corresponds to the first and second overtone regions of the NIR spectrum (Figure 3D). 

**Figure 4:**
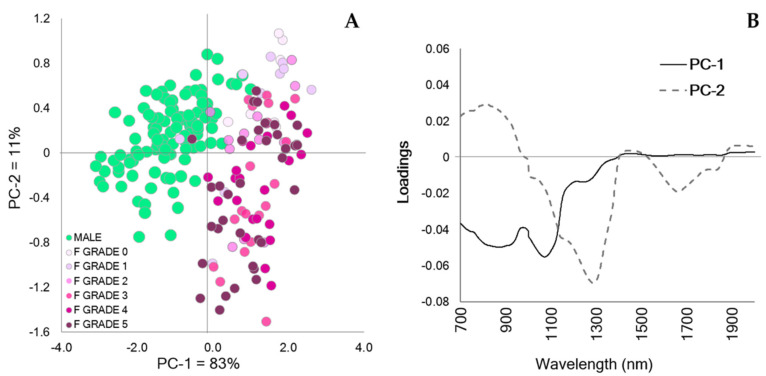
Principal component analysis (PCA) of the transformed NIR spectra (700–2000 nm) generated from the calibration dataset. (**A**) The PCA scores plot indicates a distinct pattern between *A. houstonensis* males (M) and females (F). Males are depicted as green dots, and females are color-coded according to their ultrasound grade (0–5; 0 indicating lack of follicular development and 5 indicating advanced follicular development). Two factors explained 94% of the database variance contributing to biological sex in *A. houstonensis*. (**B**) PCA loadings (700–2000 nm) highlighting the dominant peaks that explained the trends presented in the scores plot (4A): PC-1 = 83% and PC-2 = 11%.

The PCA scores plot (Figure 4A) revealed separation between the sexes, with the majority of males located in the negative quadrants of principal component (PC) 1 and the females located across the positive quadrants. Additionally, distinct trends were observed for females of varying ultrasound grades (discussed below). The spectral differences were dominated by the peaks found in the positive and negative direction of the PCA loadings (Figure 4B), where the first two PCs explained 94% of the variation in the spectral database. The Hotelling’s T^2^ influence plot generated by the PCA indicated that no outliers were present in the spectral dataset. 

Post-hoc examination of the PCA scores plot, including ultrasound grades, indicated that the spectra of females exhibiting low ultrasound grades (0–2; corresponding to non-gravid or low gravidity) were clustered within the positive quadrant of PC-2, while females exhibiting high ultrasound grades (3–5; moderate to advanced gravidity) were present across both the positive and negative quadrants of PC-2. Furthermore, four out of 36 females in the external validation dataset were misclassified as males, with two of these individuals being non-gravid females and the other two being gravid females. 

**Figure 5:**
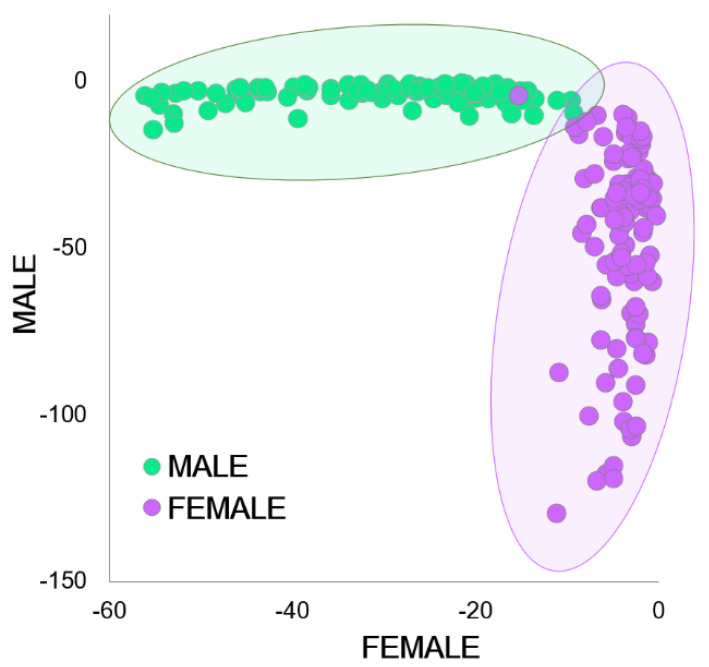
Linear discriminant analysis (PCA-LDA) plot indicating distinct trends for the transformed NIR spectra (700–2000 nm) collected from male and female *A. houstonensis* individuals.

After applying the top-down approach for PC selection in the PCA-LDA, three PCs were chosen, which explained >98% of the database variance. The percentages of accurately classified sample spectra generated by the confusion matrix can be found in Table 1. Spectra from the cloacal region of *A. houstonensis* were accurately classified in 99.1 ± 0.0%, 98.1 ± 2.6%, and 94.0 ± 8.5% of the calibration, internal validation, and external validation datasets, respectively. The high percentage of classification between both sexes was also observed in the PCA-LDA plot (Figure 5).

## 4. Discussion

The PCA-LDA prediction model successfully classified *A. houstonensis* biological sex with accuracy higher than 90%, suggesting that NIRS is an effective tool for assessing this parameter in a living animal system. Spectral signatures of females with low ultrasound grades (i.e., early egg development) were clustered in close proximity to spectra from males, which may have contributed to their misclassification as males in the validation process; overall, we observed that non-gravid females were more likely to be classified as males compared to gravid females, indicating that males and non-gravid females are less dissimilar than males and gravid females. Altogether, these data reveal that not only do male and female *A. houstonensis* differ with regard to their chemical profiles, but females of varying follicular developmental stages also appear distinguishable from one another, such that females trend further away from males in their chemical spectra as they progress in follicular state. The ability of NIRS to accurately detect biochemical differences between sexes as well as in females with varying degrees of gravidity provides novel insight into their underlying reproductive state. Previous research in mammal species, including red deer (*Cervus elaphus*), fallow deer (*Dama dama*), giant pandas (*Ailuropoda melanoleuca*), alpacas (*Lama pacos*), and several livestock species, has also demonstrated the utility of NIRS as an analytical technique to assist in the management decisions surrounding breeding programs [35,36,37,38,39]. One major difference between applications of NIRS in mammals versus in amphibians is that mammal studies require the collection of biological samples, such as feces, hair, or blood plasma, to conduct analyses; on the other hand, as shown here, NIR spectra can be collected directly from the skin of live amphibians. 

Although biological sex identification is possible through the observance of sex-specific morphological traits and breeding behaviors in many adult amphibian species, such traits may be absent or imperceptible in sexually monomorphic species, immature juveniles, small animals, and individuals outside of the mating season [11]. As opposed to gross morphological traits, the information gleaned by NIRS comprises chemical signals, including changes in endocrinology and chemical communication through pheromones, that may be present even when sex-specific anatomical traits are absent. Once a calibration model has been tested and shown to reliably discriminate spectra for the variable of interest, analysis of future spectral scans can be performed rapidly and reliably [40]. NIRS differs from other sex discrimination techniques such as ultrasonography, where assessing biological sex requires the female being sampled to be sexually mature and at a stage in egg development where follicles are both present and sufficient in size to be detected [41]. Additionally, interpretation of ultrasound images requires extensive species-specific knowledge and may not translate well across species or genera [18]. Another advantage of NIRS is that the probe sizes available for spectral data collection are much smaller (e.g., 5 mm diameter) compared to the smallest ultrasound probes (e.g., 2 cm diameter) available. This allows NIRS to be applied in applications where smaller-bodied individuals are concerned (e.g., two-lined salamander, *Eurycea cirrigera*, Burger, pers comm). Additionally, exogenous hormone-induced gametogenesis is useful for determining biological sex, but it can only be applied to sexually mature individuals and has the potential to result in nonresponders [11] (i.e., non-release of sperm or eggs). Furthermore, the hormone regimens used to induce spermiation or ovulation are often sex-specific and therefore may not be feasible for application in individuals of unknown sex [10,42]. Although ultrasonography and hormone-induced gamete expression are often needed to validate the sex of an individual, NIRS may serve as an alternative methodology once a prediction model has been established. Overall, this study highlights NIRS as a novel technique that is as effective as traditional methods used for sex discrimination, without the added expense of chemical reagents, collection time, and dependence on reproductive maturation that traditional techniques rely on. 

Preliminary results from our lab demonstrating NIRS as a novel method to discriminate sex in amphibians have thus far been evaluated in a limited number of caudate and anuran species [11,31,43]. For caudate species, sex has been classified with accuracy of 69.4% in northern dusky salamanders (*Desmognathus fuscus*), 100% in tiger salamanders (*Ambystoma tigrinum*), and 92.7% in giant Chinese salamanders (*Andrias davidianus*) for the external validation process [31,43]. The use of NIRS to classify sex in anurans has also been examined, with preliminary results indicating accuracy of 94.5% in Mississippi gopher frogs (*Lithobates sevosa*) [44], 98.4% in Fowler’s toads (*Anaxyrus fowleri*), and 97.5% in Colorado boreal toads (*Anaxyrus boreas*) [11]. Although the specifics of chemosensory communication are not well understood in anurans, it is known that anurans produce and possess the chemosignals and neural receptors necessary for processing pheromone peptides and proteins [32,45]. It is therefore possible that sex-specific differences in the chemical signals produced by these glands may drive the differences observed in their respective spectral signatures. This study serves as the first comprehensive investigation of NIRS as a highly effective and non-invasive technique for sex discrimination and gravidity in a model anuran species. 

Future research should investigate whether NIRS can be used on more species as a sex-discriminatory tool, especially for monomorphic amphibian species, immature individuals, and individuals outside of the breeding season who do not exhibit sex-specific traits. For example, numerous amphibians, such as leiopelmatid frogs (*Leiopelma pakeka*) and hellbender salamanders (*Cryptobranchus alleganiensis*), exhibit no reliable indicators of sexual dimorphism prior to sexual maturity, which may take several years to reach and even then may only be expressed during their respective mating seasons [46,47,48]. 

## 5. Conclusions

In this study, we have demonstrated the potential of NIRS as a biophotonic technique to capture chemical signals in living amphibians to effectively discriminate biological sex and gravidity. These findings can be further applied to other threatened amphibians whose sex is ambiguous and difficult to discern (e.g., juveniles and monomorphic species) or for evaluating the sexual maturation of females. Considering that amphibians represent the most threatened class of vertebrates, methods to detect and assess crucial physiological information are needed to effectively inform the management decisions concerning at-risk populations. As NIRS is a non-invasive, cost-effective tool capable of generating a robust throughput of chemical information, it may benefit wildlife conservation managers to partner with experts in specific management situations where knowledge of physiological factors (e.g., biological sex) is needed to make reliable, on-the-ground decisions. 

## Figures and Tables

**Table 1:** Linear discriminant analysis (PCA-LDA) spectra classification and % of accuracy for detecting biological sex in *A. houstonensis* using three PCs.

Category	# Spectra	Cal 80%	Val 20%	Ext Val
Female	243	107/108 (99.1%)	26/27 (96.3%)	95/108 (88.0%)
Male	153	107/108 (99.1%)	27/27 (100.0%)	18/18 (100.0%)
Mean ± SD	396	99.1 ± 0.0%	98.1 ± 2.6%	94.0 ± 8.5%

## Data Availability

Data are available upon request from Carrie K. Vance (ckv7@msstate.edu).
